# Cannabinoid Receptor CB_2_ Modulates Axon Guidance

**DOI:** 10.1371/journal.pone.0070849

**Published:** 2013-08-09

**Authors:** Gabriel Duff, Anteneh Argaw, Bruno Cecyre, Hosni Cherif, Nicolas Tea, Nawal Zabouri, Christian Casanova, Maurice Ptito, Jean-François Bouchard

**Affiliations:** 1 School of Optometry, University of Montreal, Montreal, Quebec, Canada; 2 Faculty of Pharmacy, University of Montreal, Montreal, Quebec, Canada; 3 Department of Biomedical Science, Faculty of Medicine, University of Montreal, Montreal, Quebec, Canada; Institut de la Vision, France

## Abstract

Navigation of retinal projections towards their targets is regulated by guidance molecules and growth cone transduction mechanisms. Here, we present *in vitro* and *in vivo* evidences that the cannabinoid receptor 2 (CB_2_R) is expressed along the retino-thalamic pathway and exerts a modulatory action on axon guidance. These effects are specific to CB_2_R since no changes were observed in mice where the gene coding for this receptor was altered (*cnr2* ^−/−^). The CB_2_R induced morphological changes observed at the growth cone are PKA dependent and require the presence of the netrin-1 receptor, Deleted in Colorectal Cancer. Interfering with endogenous CB_2_R signalling using pharmacological agents increased retinal axon length and induced aberrant projections. Additionally, *cnr2* ^−/−^ mice showed abnormal eye-specific segregation of retinal projections in the dorsal lateral geniculate nucleus (dLGN) indicating CB_2_R’s implication in retinothalamic development. Overall, this study demonstrates that the contribution of endocannabinoids to brain development is not solely mediated by CB_1_R, but also involves CB_2_R.

## Introduction

The endogenous cannabinoid system comprises the endocannabinoids (eCBs), the enzymes involved in their synthesis and degradation, and their receptors [1]. Type 1 (CB_1_R) and type 2 (CB_2_R) are the principal receptors characterized so far [2]. CB_2_R was first observed in peripheral and immune tissues [3] and there are increasing evidences that it is also expressed in neurons. For example, CB_2_Rs are found in mouse cerebellum [4] as well as in rat dorsal root ganglia neurons and neuronal progenitors [5], [6]. The presence of CB_2_R was also reported in brainstem, cerebellar and hippocampal pyramidal neurons of adult mammals [7], [8]. Moreover, this receptor [9] and its mRNA [10] have also been reported in the adult rat retina. There is no evidence as yet of its expression in the developing neurovisual system.

In 2000, Fernandez-Ruiz *et al* proposed that the eCB system is involved in numerous processes regulating the development of the Central Nervous System (CNS) [11]. eCBs, through CB_1_R, modulate pyramidal cell progenitor proliferation and immature pyramidal cell migration. Furthermore, CB_1_R deletion causes deficits in pyramidal cell fasciculation [12]. Deficiencies in fasciculation and axonal growth have also been reported following pharmacological activation of CB_1_R in chick, and gene knockdown in zebrafish [13]. Its role in axon guidance has been shown in GABAergic interneurons, where its activation induced growth cone collapse resulting in chemorepulsion [14]. Recently, we demonstrated that CB_1_R modulates retinal projection axon guidance and development [15]. Although the implication of CB_2_R in proliferation, differentiation and survival of neuronal cells is well documented [16]–[18], no emphasis has been put on its putative role on axon guidance during CNS development. Because eCB levels fluctuate in the brain during development [19] and since the presence of CB_2_R has been reported in the developing CNS, it is plausible to speculate that eCBs, via CB_2_R, affect axonal navigation.

During the development of the visual system, Retinal Ganglion Cell (RGC) axons navigate from the retina to their thalamic and midbrain targets. In rodents, they steer towards the optic chiasm where the majority of axons decussate to reach the contralateral side while a small contingent remains ipsilaterally. When axons reach their main targets, namely the dLGN and the superior colliculus (SC), they form synaptic connections [20].

Our study indicates that the pharmacological and genetic manipulations of CB_2_R activity affect RGC growth and retinothalamic development. Importantly and similar to CB_1_R, CB_2_R-induced reorganization of the growth cone implicates the cAMP/PKA pathway and the DCC receptor. The present study is the first demonstration that CB_2_R is expressed in the developping visual system and that it plays a role in axon guidance and brain wiring.

## Materials and Methods

Protocols for animal experimentation were approved by the *Comité de déontologie de l’expérimentation sur les animaux* of the University of Montreal (Permit numbers: 12–071, 12–080, and 12–081) and handled in accordance to the Canadian Council on Animal Care recommendations.

### Reagents

Antibody raised against GAPDH, Bovine serum albumin (BSA), ciliary neurotrophic factor (CNTF), dibutyryl cAMP (db-cAMP), DNase, dextran-FITC, forskolin (FSK), Hoechst 33258, insulin, KT5720, laminin, rabbit polyclonal anti-CB1R, monoclonal anti-β-actin, monoclonal anti-MAP Kinase (Diphosphorylated Erk-1&2), poly-D-lysine, progesterone, putrescine, selenium, apo-transferrin, triiodo-thyronine, and trypsin were purchased from Sigma (Oakville, ON). Rabbit anti-mouse macrophage and mouse anti-Thy-1.2 monoclonal IgM (µ chain specific) were obtained from Accurate Chemical (Westbury, NY). B27, Dulbecco's Phosphate-Buffered Saline (DPBS), Fetal Bovine Serum (FBS), glutamine, N2, neurobasal media, penicillin-streptomycin, S-MEM and sodium pyruvate were bought from Invitrogen Canada (Burlington, ON). Antibodies directed against NCAM, neurofilament-L, p-AKT (ser473), AKT, p-S6 (ser235/236), S6, p-PKA C (thr197), and PKA C-α were from Cell Signaling Tech (Beverly, MA). Normal donkey serum (NDS) and normal goat serum (NGS) were purchased from Jackson Immuno (West Grove, PA). Shandon ImmuMount was bought from Thermo Scientific (Pittsburgh, PA). AM630, JTE907, JWH015, and JWH133 were acquired from Tocris Bioscience (Ellisville, MI). Primary antibodies raised against Brn3a, GAP-43, p-ERK1/2, ERK1/2, and cAMP were from Chemicon International (Temecula, CA). Monoclonal DCC antibodies against extracellular (DCC_EX_, G92-13) or intracellular (DCC_IN_, G97–449) epitopes of DCC were obtained from PharMingen (Mississauga, ON, Canada). Anti-DCC_FB_ AF5, H89, LNAC, LY294002 and rapamycin were purchased from EMD (La Jolla, CA). Primary antibody against L1 and alexa fluor conjugated secondary antibodies (Alexa-488 and Alexa-546) were obtained from Invitrogen. Avidin-biotin-peroxidase complex ABC Kit, 3,3′-diaminobenzidine tetrahydrochloride (DAB)-Nickel, and donkey anti-goat biotinylated secondary antibody were from Vector Labs (Burlingame, CA). The B fragment of the cholera toxin (CTb) and goat anti-CTb were from List Biological Laboratories (Campbell, CA). Rabbit polyclonal anti-CB_2_R, its blocking peptide (human CB_2_R amino acid sequence 20–33 (NPMKDYMILSGPQK)), and rabbit polyclonal anti-MGL were purchased from Cayman (Ann Arbor, Michigan). Goat polyclonal anti-CB_2_R was obtained from Santa Cruz Biotechnology (Santa Cruz, CA). The monoclonal anti-Netrin 1 (MAB1109) was purchased from R&D Systems (Minneapolis, MN) and the polyclonal anti-Netrin 1 (PN2) was kindly provided by Pr. Timothy Kennedy (Montreal Neurological Institute, Montreal, QC). Anti-NAPE-PLD and anti-DAGLα were kind gifts from Ken Mackie (Department of Psychological & Brain Sciences, Indiana University, Bloomington, IN).

### Purified Retinal Ganglion Cell Culture

Retinal ganglion cells (RGC) from P7-P8 mice (Charles River, St-Constant, QC) were purified and cultured according to a protocol previously described by Barres *et al*. [21]. In brief, following enucleation, retinas were dissected and enzymatically dissociated, at 37°C for 30 min, in a papain solution (15 U/ml in DPBS) containing 1 mM L-cysteine. The retinas were then triturated sequentially, with a 1 ml pipette, in a solution containing ovomucoid (1.5 mg/ml), DNase (0.004%), BSA (1.5 mg/ml) and rabbit antibodies directed against mouse-macrophage (1∶75) to yield a suspension of single cells. The suspension was then centrifuged and washed in a high concentration ovomucoid-BSA solution (10 mg/ml for each in DPBS). The dissociated cells were resuspended in DPBS containing BSA (0.2 mg/ml) and insulin (5 µg/ml).

RGCs were purified using the two-step pαnning procedure [21], [22]. Briefly, to remove macrophages, the retinal suspension was incubated at room temperature in petri dishes coated with affinity-purified goat anti-rabbit IgG (H+L). The nonadherent cells were then transferred to a petri dish that had been coated with affinity purified goat anti-mouse IgM (µ chain specific) followed by anti-Thy-1.2 monoclonal IgM. The adherent RGCs were first released enzymatically by incubating them in a 0.125% trypsin solution at 37°C and 5% CO_2_ followed by manually pipetting an enzyme inhibitor solution (30% FBS in Neurobasal) along the surface of the dish.

Purified RGCs were plated on poly-D-lysine (10 µg/ml) and laminin (5 µg/ml) coated glass coverslips (number 0 Deckgläser; Carolina Biological, Burlington, NC) in 24-well plates. RGCs were cultured in 600 µl of serum-free medium modified from Bottenstein and Sato [23]. Neurobasal media was supplemented with B27, selenium, putrescine, triiodo-thyronine, transferrin, progesterone, pyruvate (1 mM), glutamine (2 mM), ciliary neurotrophic factor (CNTF; 10 ng/ml), brain-derived neurotrophic factor (BDNF; 50 ng/ml), insulin (5 µg/ml), and FSK (10 µM). RGCs were cultured at 37°C and 5% CO_2_.

### Primary Neuron Culture

Staged pregnant mice were obtained from Charles River (St-Constant, QC). E14–15 mouse embryo cortices were isolated surgically and transferred in a vial containing 2 ml S-MEM at 37°C supplemented with 2.5% trypsin and 2 mg/ml DNase for 15 minutes. The pellet was then transferred into 10 ml S-MEM with cold 10% FBS and stored at 4°C. Following centrifugation, pellet was transferred in 2 ml S-MEM with 10% FBS and triturated 3 or 4 times to yield to a suspension of single neurons. Then, 10 ml of Neurobasal medium was added to this suspension. Dissociated cells were counted and plated on 12 mm poly-D-lysine treated glass coverslips (20 µg/ml; 50 000 cells/well). Cells were cultured for 2 days *in vitro* (DIV2) in Neurobasal medium containing 1% B-27, 100 U/ml penicillin, 100 µg/ml streptomycin, 0.25% N2 and 0.5 mM glutamine for growth cone analysis. Then, neurons were treated with, either CB_2_R agonists (300 nM JWH133 or JWH015), CB_2_R inverse agonists (300 nM AM630 or JTE907), adenylate cyclase activator (10 µM FSK), PKA inhibitors (200 nM KT5720 or 2 µM H89) or DCC function blocking (3.5 µg/ml anti-DCC_FB_ AF5) for 1 hour for growth cone morphology experiments or for 15 minutes for cAMP immunocytochemistry.

### Retinal Explant Culture

E14–15 mouse embryo retinas were isolated and dissected in small segments in ice cold DPBS and plated in 24 well plates on 12 mm poly-D-Lysine (20 µg/ml) and laminin (5 µg/ml) treated glass coverslips. Explants were cultured in Neurobasal supplemented with 100 U/ml penicillin, 100 µg/ml streptomycin, 5 µg/ml LNAC, 1% B27, 40 ng/ml selenium, 16 µg/ml putrescine, 0.04 ng/ml triiodo-thyronine, 100 µg/ml transferrin, 60 ng/ml progesterone, 100 µg/ml BSA, 1 mM sodium pyruvate, 2 mM glutamine, 10 ng/ml ciliary neurotrophic factor (CNTF), 5 µg/ml insulin, and 10 µM FSK. Explants were treated for 15 hours at DIV0 (1 hour following platting) for outgrowth analysis or for 1 hour at DIV1 for growth cone analysis assay. Photomicrographs for outgrowth analysis were taken using an Olympus BX51WI microscope (Olympus Canada, Markham, ON) with a 10X objective lens and analyzed using Image Pro Plus 5.1 software (Media Cybernetics, Bethesda, MD). The total length of axon bundles was quantified and expressed as mean ± SEM. Statistical significance of differences between means was evaluated by analysis of variance (ANOVA) with Bonferroni’s *post hoc* test (Systat).

### Immunocytochemistry

Plates were washed with cold PBS (pH 7.4) and fixed in 4% paraformaldehyde in PBS for 10 minutes. Primary neuron and retinal explants cultures were blocked in 2% NGS and 2% BSA in PBS during 30 minutes at room temperature. Antibodies were added overnight in a blocking solution at the following concentrations: anti-GAP-43 1:1,000, anti-CB_2_Rsc 1∶100, anti-CB_2_Rcayman 1∶500, anti-MGL 1∶500, anti-L1 1:500, anti-cAMP 1∶1,000, anti-β-actin 1∶1,000, anti-NFL 1∶500, anti-NAPE-PLD 1∶200, anti-DAGLα 1∶200, anti-DCC_IN_ 1∶500. The following day, the neurons or explants were washed with PBS-tween (PBST), incubated with secondary antibodies Alexa 488 or 546 for 2 hours at room temperature. Nuclei were labeled with Hoechst 33258 and coverslips were mounted with ImmuMount (Thermo Scientific, Pittsburgh, PA).

### Quantification of cAMP Immunoreactivity

All photomicrographs used for quantification were taken using an inverted Olympus IX71 microscope (Olympus Canada, Markham, ON) with a 60X objective lens and identical exposure time to allow for comparison of measurements. Fluorescence intensity at the growth cone was corrected for background noise and quantified using Image Pro Plus 5.1 software. For growth cone analysis, both Differential Interference Contrast (DIC) and fluorescent images were taken. Fluorescence intensity per squared micrometer is expressed as the mean ± SEM. Statistical significance was evaluated by analysis of variance (ANOVA) with Bonferroni’s *post hoc* test (Systat).

### Western Blots

Hamster pups were sacrificed at various ages, namely: P1, 3, 5. They were deeply anesthetized by hypothermia. Eyes were immediately removed for Western blot analysis. The retinas were dissected on ice, homogenized by hand using a sterile pestle in RIPA lysis buffer, supplemented with a protease inhibitor mixture (aprotinin, leupeptin, pepstatin, and phenylmethylsulfonyl fluoride (PMSF)). Samples were then centrifuged at 13,000 rpm at 4°C for 10 min and the supernatant was extracted and stored. Protein contents were equalized using a BCA Protein Assay kit (Thermo Scientific, Fischer scientific, Ottawa, ON). In another set of experiments, dissociated mouse primary neurons were cultured for 2 DIVs at a density of approximately 250,000 cells/dish in 35 mm poly-D-lysine coated dish. After 10-minute treatment with CB_2_R agonists (300 nM JWH133 or JWH015), CB_2_R inverse agonists (300 nM AM630 or JTE907), or adenylate cyclase activator (10 µM FSK), neurons were washed once with ice-cold PBS and lysed with Laemmli sample buffer. Western blot analysis was performed using anti-CB_2_Rcayman 1∶1,000, anti-CB_1_R 1∶1,000, anti-β-actin 1∶5,000, anti-GAPDH 1∶20,000, anti-p-PKA C 1∶1,000, anti-PKA Cα 1∶1,000 anti-DCC_IN_ 1∶2,000, anti-NCAM 1∶5,000, anti-ERK1/2 1:5,000, anti-p-ERK1/2 1:2,000, anti-AKT 1∶1,000, anti-p-AKT 1∶1,000, anti-S6 1:2,000, anti-p-S6 1:2,000 overnight at 4°C. Results were visualized using the Western Lighting Chemiluminescence Reagent Plus kit (Perkin-Elmer, Boston, MA, USA). Immunoreactivity was imaged with a ScanJet 5300C (Hewlett Packard Canada, Mississauga, ON, Canada).

### Surface Biotinylation

E14–15 neurons were plated and cultured for 2 days at a density of 2,000,000 cells per 100 mm PDL-coated tissue culture dish. On day 2, cells were treated with CB_2_R inverse agonists (300nM AM630 or JTE907), PKA inhibitors (200 nM KT5720 or 2 µM H89), adenylate cyclase activator (10 µM FSK), or vehicle for 15 min. Neurons were then washed with ice-cold PBS containing 0.1 mM calcium chloride and 1 mM magnesium chloride, pH 7.4, to halt protein trafficking. Surface biotinylation was performed by adding EZ-Link Sulfo-NHS-LC-biotin (Thermo Scientific, Rockford, IL), 5 ml per plate at 0.5 mg/ml in PBS at 4°C for 30 min, removed, and the reaction was quenched by the addition of 5 ml of 10 mM ice-cold glycine in PBS at 4°C for two 10 min periods. Subsequently, neurons were washed twice with 5 ml of ice-cold PBS and lysed with RIPA buffer. Biotinylated proteins were precipitated with streptavidin–agarose (Thermo Scientific) and analyzed by Western blot.

### Growth Cone Behavior Assay

Retinal explants were cultured in borosilicate-chambered coverglass (Lab-Tek; Rochester, NY). At DIV1, explants were installed in a Live Cell chamber (5% CO_2_, 37°C) (Neue Bioscience, Camp Hill, PA) mounted to an inverted Olympus IX71 microscope. Glass micropipettes with an orifice of 2–3 µm diameter were positionned at 45° and 100 µm away from the growth cone of interest. A concentration gradient was created using a micro-injector (Picoplus, Harvard Apparatus - Model 702213).

### Intraocular Injections

Syrian golden hamsters (Charles River, St-Constant, QC) were used for intraocular injections. These mammals are born with a relatively premature nervous system [24]. Compared with rats and mice, hamsters have a shorter gestation period. The gestation periods are 21.5, 18.5 and 15.5 days for rats, mice and hamsters, respectively [24]. The neural events that characterize the development of the mouse and hamster nervous system, including the neuro-visual system, occur at almost identical time points of embryonic development [24]. For example, RGC generation starts at E9.5 for hamsters and E10.5 for mice while the dLGN starts to develop at E10.5 for both models [24], [25]. At birth (postnatal day 0, P0), RGC axons have not all reached their thalamic and midbrain targets in hamster. By P3, virtually all RGC axons have reached their targets [26]. To take advantage of this opportunity, 1 day after birth (P1), hamsters received a 2 µl unilateral injection of a 1% solution of the beta subunit of the cholera toxin coupled to FITC (CTb-FITC), a highly sensitive anterograde tracer, in either 0.9% saline solution, 300 µM JWH133 (CB_2_R agonist) or 300 µM AM630, a CB_2_R inverse agonist. Briefly, under an operating microscope, a small incision was made in the eyelids to access the right eye; the injections were administered using a glass micropipette attached to a 10 µl Hamilton syringe. The micropipette was carefully inserted into the vitreous at an angle to avoid damaging the lens. Following the injection, the eyelids were closed with surgical glue (Vetbond; 3 M, St-Paul, MN). The same surgical procedures were performed using adult mice where the gene coding for the CB_2_R was genetically modified to produce non-functional CB_2_R (*cnr2* ^−/−^). In these series of experiments, adult mice (*cnr2* ^−/−^ and their wildtypes (*cnr2^+/+^*)) were injected in the right eye with the CTb-Alexa-546 and the left one received an injection of CTb-Alexa-488. Four days following the injections, animals were perfused transcardially with 0.1 M PBS followed by 4% paraformaldehyde in PBS. The brains were removed, postfixed overnight at 4°C, cryoprotected by infiltration of buffered sucrose, flash frozen and kept at −80°C until further processing.

### Immunohistochemistry

The presence of the CB_2_R during early postnatal development was investigated by immunohistochemistry. The RGCs were labeled with Brn3a. Retinal sections were washed in 0.1 M PBS, post fixed for 5 minutes in a 70% solution of ethanol, rinsed in 0.03% Triton X-100 buffered saline and blocked in 10% normal donkey serum (NDS, Jackson immunoresearch laboratories, West Grove, PA) and 0.5% Triton X-100 in buffered saline for 1 h. The sections were then co-incubated overnight in rabbit anti-CB_2_R (1∶200, Cayman) solution with a mouse anti-Brn3a. After incubation with the primary antibodies, the sections were washed in buffered saline, blocked for 30 minutes and incubated for 1 h with secondary antibodies: Alexa donkey anti-rabbit 555 for CB_2_R and Alexa donkey anti-mouse 488 for syntaxin (Molecular Probes, Eugene, OR). After washes in buffered saline, the sections were mounted with a homemade PVA-Dabco mounting medium [27]. Photomicrographs were taken using a Leica TCS SP2 laser scanning confocal microscope (Leica Microsystems, Exton, PA). Images were captured in the Alexa fluo 555/546 and Alexa fluo 488/FITC channels, pseudo-colored, merged and exported using Leica LCS software (version 2.61). The pictures were taken sequentially to ensure no ‘Bleed-through’ between channels.

The presence of the CB_2_R in the hamster neuro-visual brain (dLGN, SC, and visual cortex) was also assessed using immunohistochemistry. Forty µm thick coronal sections of tissue comprising the dLGN, SC or visual cortex were pre-incubated for 20 min at room temperature in PBS 0.1 M containing 0.3% hydrogen peroxide, followed by 1 h in PBS containing 0.3% Triton X-100 and 3% Normal Donkey Serum. The sections were then incubated for 48 h at 4°C in the blocking solution (PBS 0.3% Triton X-100 with 0.5% Donkey Serum) containing rabbit anti-CB_2_R (1∶200). Subsequently, sections were rinsed and immersed in a blocking solution for 30 min. Sections were then incubated in a blocking solution containing donkey anti-rabbit biotinylated secondary antibody (1∶200) for 2 h and then for 1 h in the avidin-biotin complex (ABC Elite). After each incubation step, rinses were carried out in PBS containing 0.3% Triton. A peroxidase-substrate kit Vector DAB-Nickel was used to develop the reaction product during a period of 4 min. Sections were then mounted onto slides, dehydrated and coverslipped with Permount. Photographs were taken with a microscope by MicroBrightField digital system (Williston, VT).

The effects of intraocular injection of CB_2_R agonist and inverse agonist were visualized by immunohistochemistry according to a protocol previously described [28]. Briefly, 40 µm thick coronal sections of tissue were incubated in 90% methanol and 0.3% H_2_O_2_ in PBS for 20 min. After several washes, they were incubated in 0.1 M glycine solution for 30 min, and then blocked overnight at 4°C. Sections were subsequently rinsed and immersed for 48 h at room temperature in a solution-containing goat 1∶4000 anti-CTb diluted in blocking solution. Afterwards, sections were rinsed and incubated 1 h with a biotinylated donkey secondary antibody directed against goat diluted in blocking solution (1∶200). Tissue was rinsed and subsequently processed using an avidin-biotin-peroxidase complex ABC Kit (1∶100) for 1 h, in the dark and at room temperature. Sections were then rinsed and preincubated in DAB/PBS for 5 min. The peroxidase was visualized by adding 0.004% H_2_O_2_ to the DAB solution for 5–10 min. Sections were finally washed five times with PBS, mounted on gelatin-chrome alum-subbed slides, air-dried, dehydrated in ethanol, cleared in xylene, and coverslipped with Depex (EMS, Hatfield, PA). Photomicrographs were taken with an Olympus BX51WI microscope (Olympus Canada, Markham, ON) using a 10X objective lens. Images were analyzed with Image Pro Plus 5.1 software.

### Retinothalamic and Retinogeniculate Projection Analyses

Axon branch growth was quantified on consecutive photomicrographs of coronal slices of brain tissue, as described previously [28], comprising the lateral terminal nucleus. Briefly, the distance between the lateral border of the nucleus of interest and the tips of the longest axon branches was measured and normalized for interthalamic distance. Axon collateral density was also quantified for the lateral terminal nucleus using an adaptation of the Sholl technique [29]. Values are expressed as the mean ± SEM. Statistical significance means was evaluated by analysis of variance (ANOVA) with Sheffe’s post hoc test (Systat).

For eye specific segregation studies in the dLGN, *cnr2* ^−/−^and *cnr2* ^+/+^adult mice were injected with the B fragment of the cholera toxin (CTb) conjugated to Alexa -546 (CTb-546; red) into the left eye and CTb-488 (green) into the right eye (1.5–2 µl; 0.5% in sterile saline). Forty-eight hours later, brain tissue was harvested and postfixed overnight in 4% PFA, cryoprotected in 30% sucrose and then sectioned coronally at 40 µm thickness, mounted onto slides and coverslipped with Immu-Mount. Images were collected and quantified by an observer “blind” to the experimental conditions to minimize any bias. Universal gains and exposures were established for each label. Raw images of the dLGN were imported to Matlab and an area of interest comprising the dLGN was cropped excluding the ventral lateral geniculate nucleus and the intergeniculate leaflet, then the degree of left and right eye projection overlap was quantified using an established multi-threshold method of analysis [30]–[32]. This approach allows for a better analysis of overlapping regions independent of the threshold. Values are expressed as the mean ± SEM. Significance of differences between means was evaluated by student t-test analysis (Systat).

## Results

### Spatio-Temporal Localization of CB_2_R in the Developing Visual System

**Figure 1:**
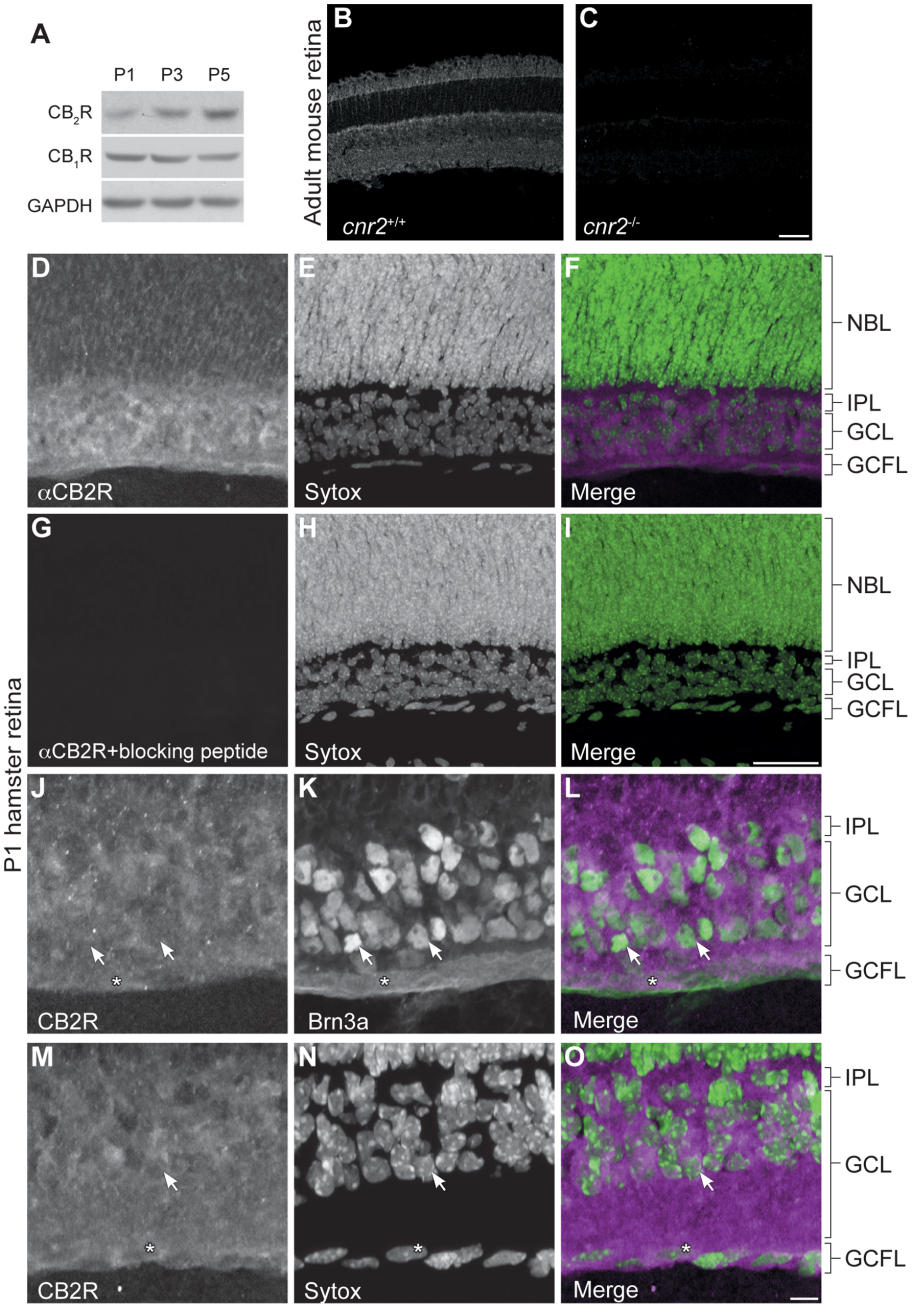
Spatio-Temporal Expression of the CB_2_R in the Retina during Postnatal Retinal Projection Development *in vivo.* (A) Western blot analysis of CB_2_R and CB_1_R expression during retinal postnatal development in the hamster. Photomicrographs illustrating the specificity of the CB_2_R antibody in the adult mouse retina (B, C) and the P1 hamster retina (D–I). (D–O), Photomicrographs of hamster retinal cross-sections showing CB_2_R (magenta) during early postnatal development (at postnatal day 1, P1). Sytox (green) was used to stain cell nuclei. Brn3a was used to label retinal ganglion cells (green). In panels (J–O), some CB2R positive retinal ganglion cell somas and fibers are indicated using arrows and asterisks respectively. NBL, Neuroblast layer; IPL, Inner plexiform layer; GCL, Ganglion cell layer; GCFL, Ganglion cell fiber layer. Specificity of the CB_2_R antibody is confimed using *cnr2* ^+/+^ and *cnr2* ^/−^ adult mouse retina. Scale bars: 50 µm (B, C); 25 µm (D–I); 10 µm (J–O).

**Figure 2:**
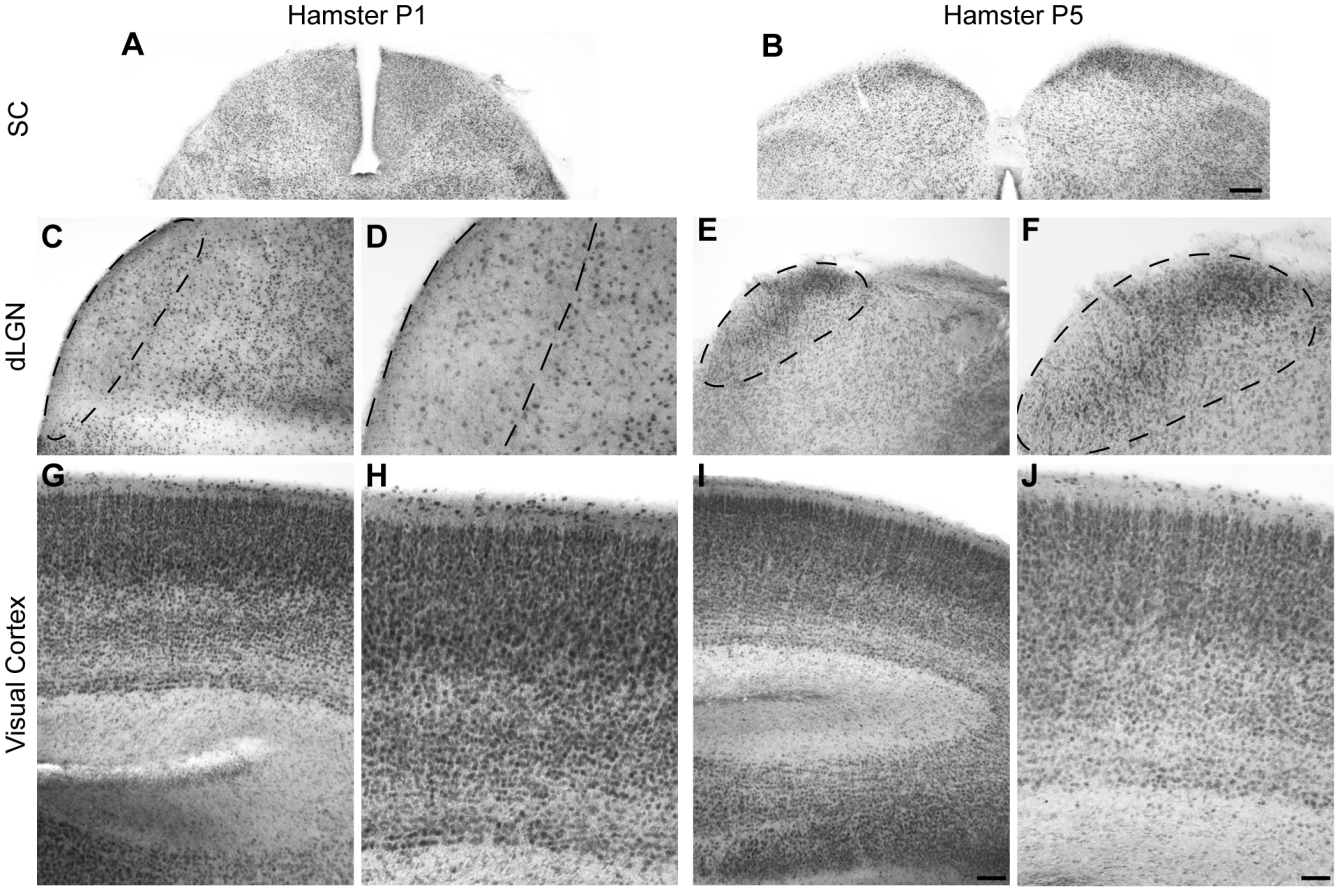
Expression of CB2R in the Superior Colliculus, dorsal Lateral Geniculate Nucleus, and Visual Cortex during Development in the Hamster. Photomicrographs of coronal sections illustrating CB_2_R expression at P1 (A, C, D, G, H) and P5 (B, E, F, I, J) in the superior colliculus (SC) (A, B), the dorsal lateral geniculate nucleus (dLGN) (C–F), and the visual cortex (G–J). In panel C–F, dLGN has been outlined for better visualization. Scale bars: 200 µm (A, B); 100 µm (C, E, G, I); 50 µm (D, F, H, J).

Despite emerging evidence supporting the presence of the eCB system in the developing CNS, the expression of CB_2_R is not well characterized in the developing neurovisual system. In order to explore the implication of CB_2_R during retinal axon guidance, we assessed its expression *in vivo*. Western blot analysis of retina lysates revealed that CB_2_R is expressed at early postnatal stages (Figure 1A). CB_2_R levels increased from postnatal day 1 *in vitro* (P1) to P5 while CB_1_R expression remained unchanged (Figure 1A). The specificity of the antibody directed against CB_2_R was tested using retinas obtained from adult *cnr2^−/−^* mice (Figure 1B–C) and P1 hamsters (Figure 1D–I). The spatio-temporal expression of CB_2_R was investigated in the RGC and RGC fiber layers (Figure 1D–0), SC (Figure 2A, B), dLGN (Figure 2C–F), and visual cortex (Figure 2G–J). During early postnatal development, this receptor is expressed in the retina, the dLGN, the SC, and the visual cortex at all ages investigated. Overall, these results demonstrate that CB_2_R is indeed found in the developing neurovisual system.

### The CB_2_R is Expressed in RGCs and their Growth Cones *In Vitro*

**Figure 3:**
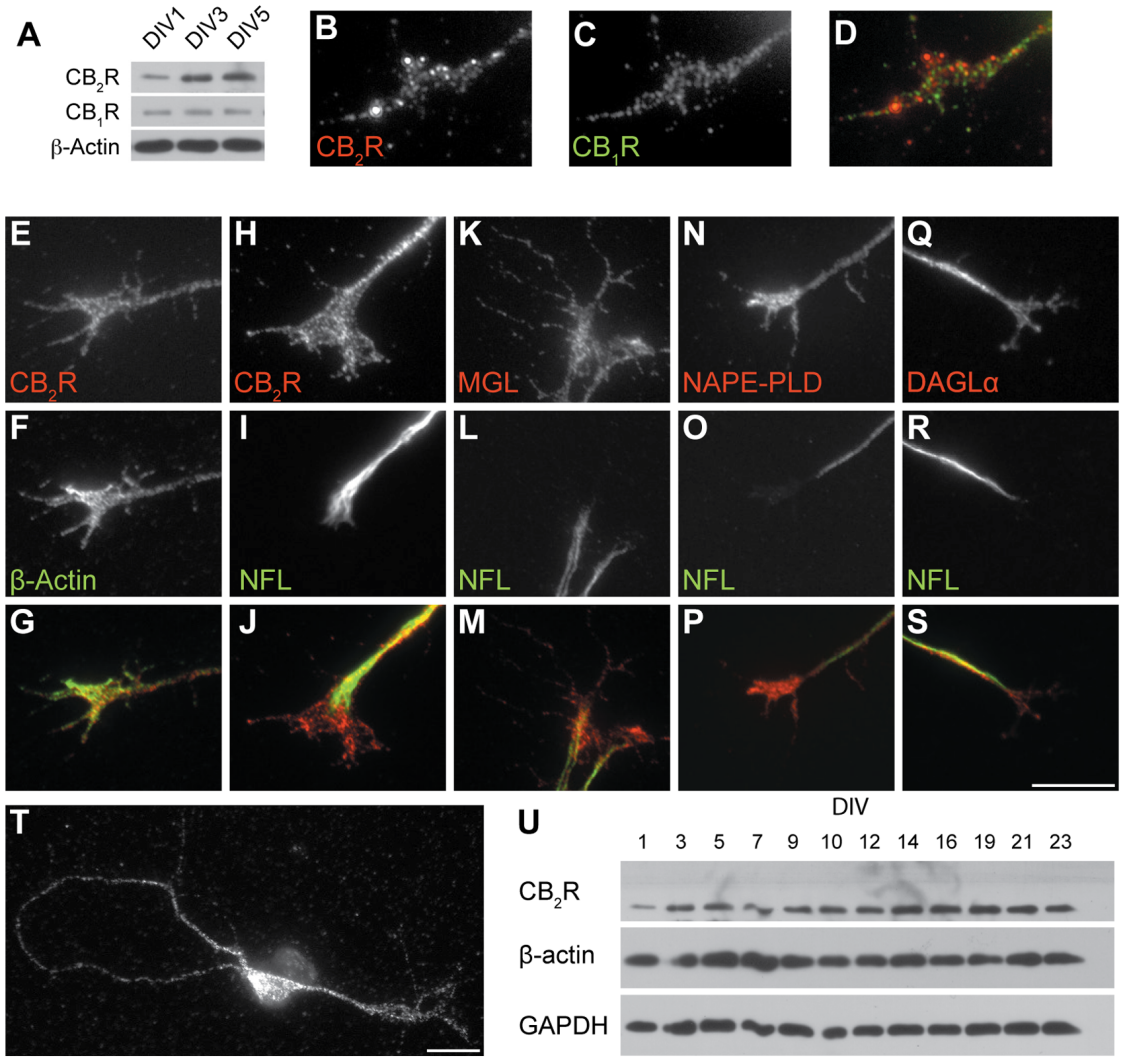
Expression of CB_2_R, CB_1_R, MGL, NAPE-PLD, and DAGLα *in vitro.* (A) Western blot shows temporal protein expression of CB_2_R and CB_1_R in cultured retinal ganglion cells. DIV1 retinal explants growth cones labeled with primary antibodies directed against CB_2_R (B, E, and H), CB_1_R (C), MGL (K), NAPE-PLD (N), and DAGLα (Q). Growth cones were also labeled for β-actin (F), Neurofilament-L (I, L, O, and R). Merged images are presented in D, G, J, M, P, and S. (T) Photomicrograph of a DIV2 primary cortical neuron immunolabeled for CB_2_R. Primary neurons were cultured for various numbers of days *in vitro* and cell extracts were equalized for total protein content. (U) Western blot showing temporal protein expression of CB_2_R, β-actin and GAPDH. Scale bars: 5 µm (B–S), 15 µm (T).

Western blot analysis of retinal ganglion cell culture lysates revealed that these neurons express CB_2_R for several DIVs (Figure 3A). Similar to what we observed *in vivo*, CB_2_R level increased from DIV1 to DIV5 while CB_1_R expression remained unchanged (Figure 3A).

The expression pattern of CB_2_R was assessed by immunocytochemistry in retinal explants obtained from mouse embryos. At DIV1, CB_2_R expression was observed in RGC neurites, growth cones, and filopodia (Figure 3B, E, H). Both CB_2_R and CB_1_R are expressed in the growth cone (Figure 3B–D). The CB_2_R immunoreactivity was also observed in primary cortical neuron cultures at DIV2 (Figure 3T). Western blot analysis of primary neuron culture lysates revealed that these neurons express CB_2_R for several DIVs (Figure 3U).

Diacylglycerol lipase α (DAGLα) and N-acyl phosphatidylethanolamine-phospholipase D (NAPE-PLD), enzymes involved in the synthesis of the main eCBs: 2-arachidonylglycerol (2-AG) and arachidonylethanolamine (AEA) respectively, as well as monoacylglycerol lipase (MGL), an enzyme implicated in the degradation of 2-AG, are also expressed in the growth cones, filopodia and neurites of RGCs (Figure 3K–S). These results demonstrate the presence of functional CB_2_Rs in retinal neurites and growth cones suggesting their implication during growth cone navigation.

### CB_2_R Reorganizes Growth Cone Morphology

**Figure 4:**
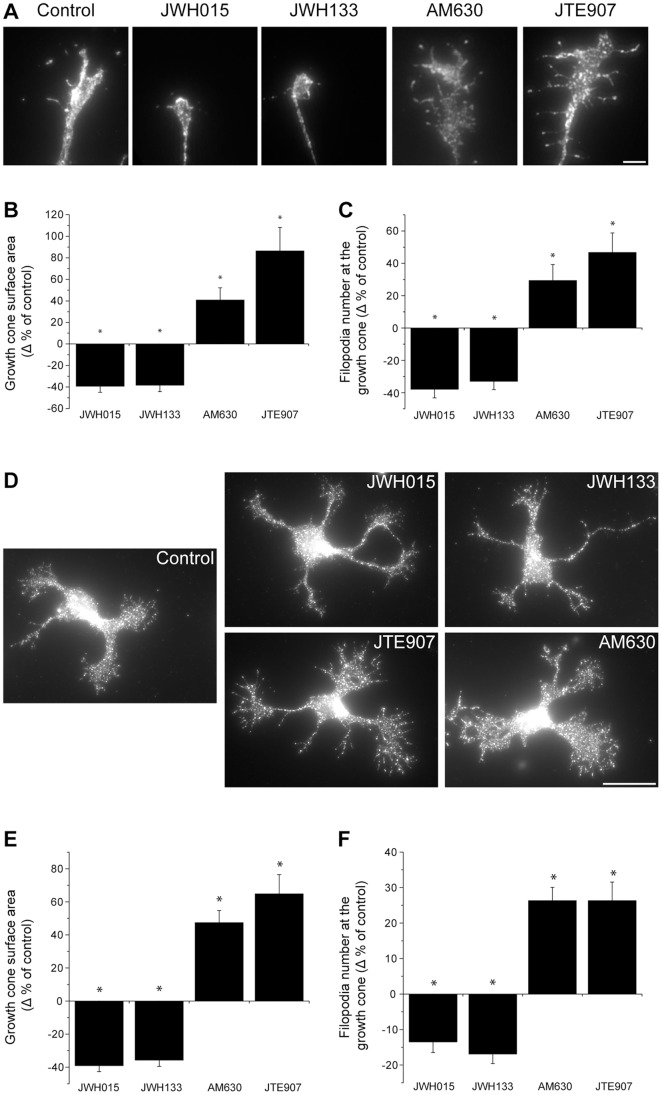
CB_2_R Reorganizes the Growth Cone Morphology. (A) Retinal explants and (D) dissociated neurons were grown for 1 and 2 days *in vitro*, respectively. Growth cones were exposed for 1 hour to 300 nM JWH015, 300 nM JWH133, 300 nM AM630, or 300 nM JTE907. Following treatment, retinal explants and neurons were fixed and immunolabeled for L1 and GAP-43, respectively. Addition of CB_2_R inverse agonists (AM630, JTE907) increased growth cone surface area (B and E) and filopodia number (C and F) while the opposite effects was observed following CB_2_R agonists (JWH015 and JWH133) treatment (mean ± SEM; n = 374 to 714 per condition). Scale bar, 5 µm (A); 20 µm (D). *P<0.05 vs control.

The implication of the CB_2_R during axon navigation was evaluated in mouse E14–15 retinal explant and primary neuron growth cones (Figure 4). Because hamsters are born with a premature nervous system, we believe that E14–15 mouse retinal explants are at similar developmental stages than a retina from a newborn hamster.

The growth cone surface area was greatly reduced when CB_2_R agonists (JWH015 or JWH133) were added to the culture; conversely, the surface area was increased following CB_2_R inverse agonist (AM630 or JTE907) stimulation (Figure 4B and E). In addition, the use of agonists caused a decrease in the number of filopodia at the growth cone, while inverse agonists increased their number (Figure 4C and F). These results demonstrate that CB_2_R regulation can directly influence growth cone morphology.

**Figure 5:**
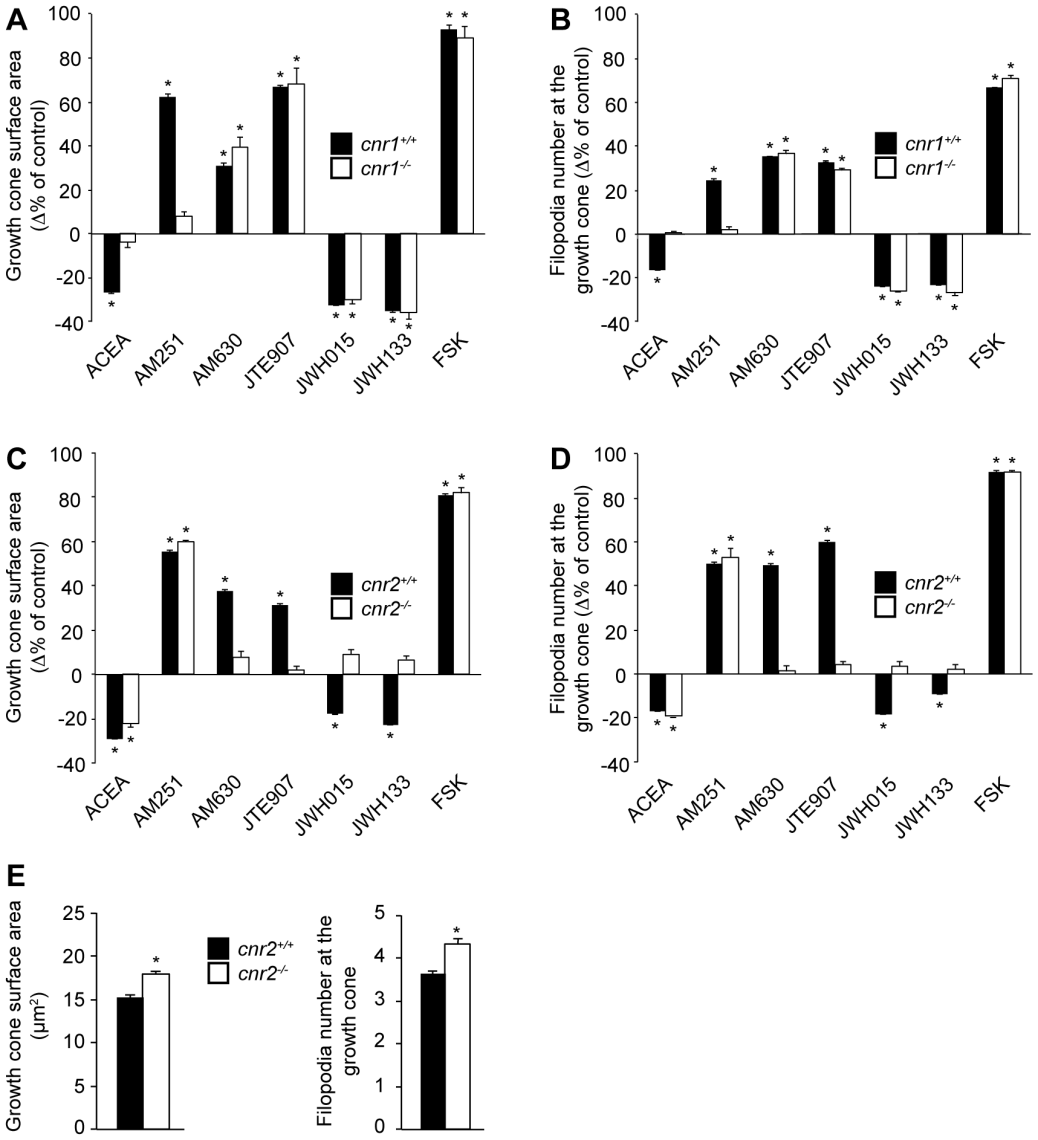
Confirmation that CB_2_R Modulates Growth Cone Morphology. Retinal explants obtained from (*cnr1^+/+^ or cnr1^−/−^*) (A and B) or (*cnr2^+/+^* or cnr2^−/−^) (C–E) embryos. In *cnr1^+/+^ or cnr2^+/+^,* administration of AM251, AM630, JTE907, and FSK increased growth cone surface area (A, C) and filopodia number (B, D) while ACEA, JWH015, and JWH133 decreased them. In *cnr1^−/−^,* responses of CB_1_R ligands (ACEA and AM251) were abolished (mean ± SEM; (A and B) n = 81 to 312 per condition, *P<0.05 vs control). In *cnr2* ^−/−^ animals, growth cone surface area (C) and filopodia number (D) were only significantly modified by ACEA, AM251 and FSK while CB_2_R ligand (JWH015, JWH133, AM630, and JTE907) stimulation did not alter these endpoints (mean ± SEM; (C and D) n = 125 to 264 per condition, *P<0.05 vs control). (E) Growth cone surface area and filopodia number were increased in *cnr2^−/−^* compared to *cnr2^+/+^.* Mean ± SEM; n = 120 to 150 per condition, *P<0.05 vs control.

Previous studies have demonstrated the implication of the CB_1_R in GABAergic interneurons and retinal axons axon development [14], [15]. Since both receptors are expressed in the developing visual system, it is possible that CB_1_R contributes to the growth cone reorganization induced by JWH015, JWH133, AM630 and JTE907. We addressed this possibility using pharmacological and genetic approaches. Addition of CB_2_R inverse agonists (AM630 and JTE907) to retinal explants obtained from *cnr1^−/−^* embryos produced a significant increase in growth cone area and filopodia number while adding CB_2_R agonists (JWH015 and JWH133) decreased growth cone surface and filopodia number (Figure 5A and B). These changes are comparable to those observed in retinal explants obtained from *cnr1^+/+^* embryos. Addition of these pharmacological agents to retinal explants derived from *cnr2^+/+^* mouse embryos induced similar effects. As predicted, these effects were completely abolished in retinal explants obtained from *cnr2^−/−^* embryos (Figure 5C and D). In another set of experiments, deletion of *cnr2* induced a significant increase in growth cone surface area and in filopodia number compare to wildtype (Figure 5E). These results confirm the contribution of the CB_2_R in the modulation of growth cone behavior. In addition, the effects of AM630, JTE907, JWH015, and JWH133 on the growth cone can be directly attributed to CB_2_R. Noteworthy, ACEA and AM251 (CB_1_R specific ligands) stimulation did not produce any effects on *cnr1* ^−/−^ growth cones (Figure 5A and B). We observed similar results in primary cortical neurons from these transgenic animals (unpublished observations). Taken together, these results demonstrate that CB_2_R influences growth cone morphology independently from CB_1_R.

### CB_2_R Modulates RGC Axon Outgrowth

**Figure 6:**
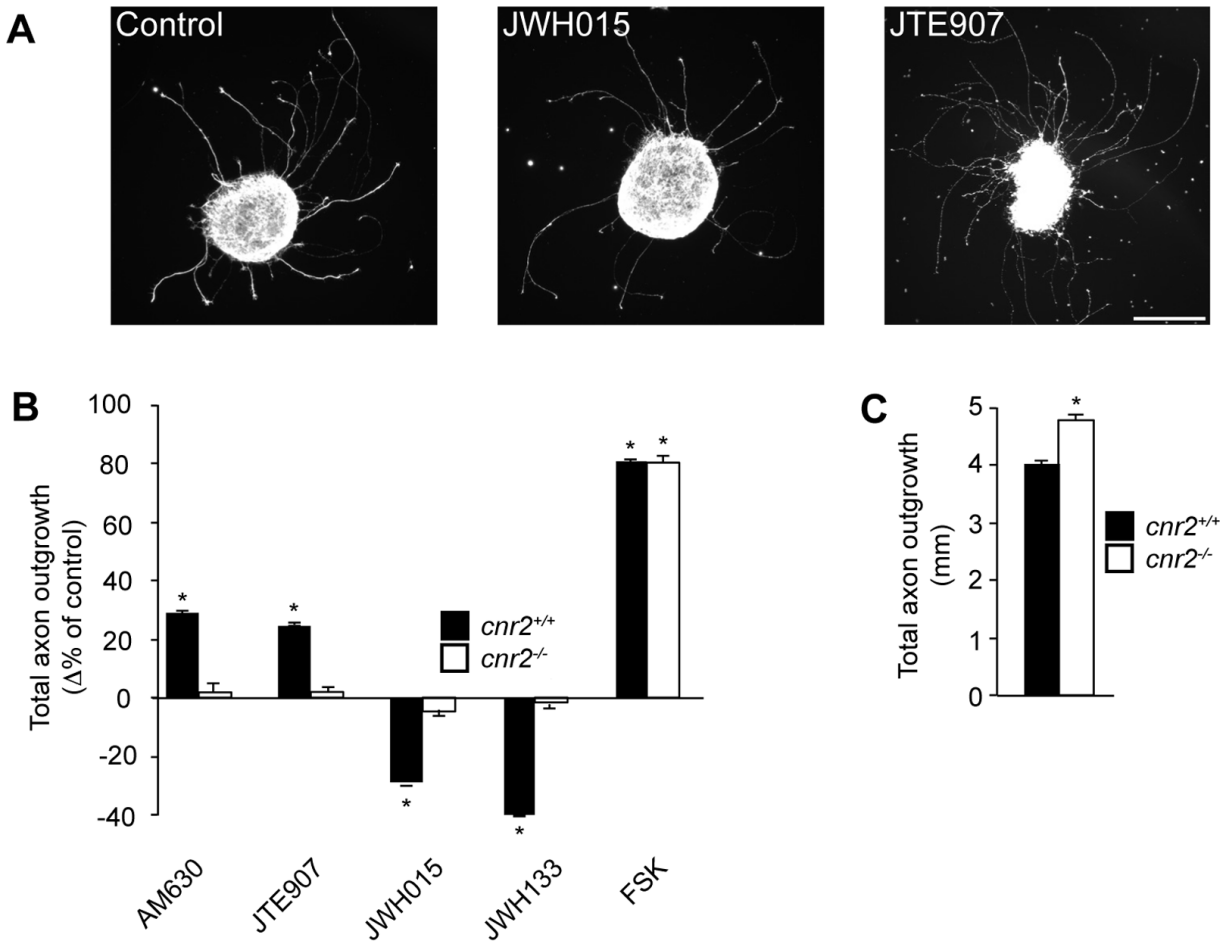
CB_2_R Modulates Axon Outgrowth. Retinal explants obtained from C57Bl/6-*cnr2* ^+/+^ or C57Bl/6-*cnr2* ^−/−^ mice were treated for 15 hours with 300 nM JWH015, 300 nM JWH133, 300 nM AM630 or 300 nM JTE907. Explants were labeled using anti-L1. (A). Representative explants obtained from *cnr2^+/+^* mice. Scale bar, 200 µm. (B) Quantification of total axon outgrowth was normalized for explant area and expressed as percentage of the control group (mean ± SEM; n = 20 to 129 explants per condition). Addition of CB_2_R agonists (JWH015 and JWH133) to cnr2^+/+^ retinal explants decreased axon outgrowth, while treatment with CB_2_R inverse agonists (AM630 and JTE907) increased it. Pharmacological modulation of CB_2_R did not induce any significant changes in explant axon outgrowth obtained from *cnr2* ^−/−^ mouse embryos. *P<0.05 vs control. (C) Under control conditions, deletion of *cnr2* signficantly increased axon outgrowth. *P<0.05 vs *cnr2* ^+/+^ (mean ± SEM; n = 75 to 129 explants per condition).

To assess whether CB_2_R could affect axonal growth, E14–15 mouse retinal explants were exposed to CB_2_R agonists or inverse agonists for 15 hours at DIV0 (Figure 6A). Treatment with CB_2_R agonists (JWH015 or JWH133) reduced total projection length of the explants whereas addition of CB_2_R inverse agonists (AM630 or JTE907) increased it (Figure 6B). Retinal fibers emerging from the explants were labeled with L1 antibody to ensure that all the neurites quantified were RGC axons since they are the only neurons that express this protein in the mouse retina [33]. These results demonstrate that CB_2_R influences RGC fiber extension. To ascertain that these effects were CB_2_R specific, we performed the same experiment using *cnr2* ^−/−^ retinal explants. In contrast to *cnr2* ^+/+^ cultures, *cnr2* ^−/−^ retinas did not demonstrate significant changes in projection length following CB_2_R agonist and inverse agonist treatment (Figure 6B). This indicates that these pharmacological agents are selective for the CB_2_R and that this receptor modulates retinal axon growth *in vitro*. Interestingly, we found an increase in axon outgrowth in retinal explants obtained from cnr2^−/−^ embryos (Figure 6C). This confirms our pharmacological results showing that CB2R inhibits axon outgrowth.

### CB_2_R Agonists Modulate Growth Cone Behaviour

**Figure 7:**
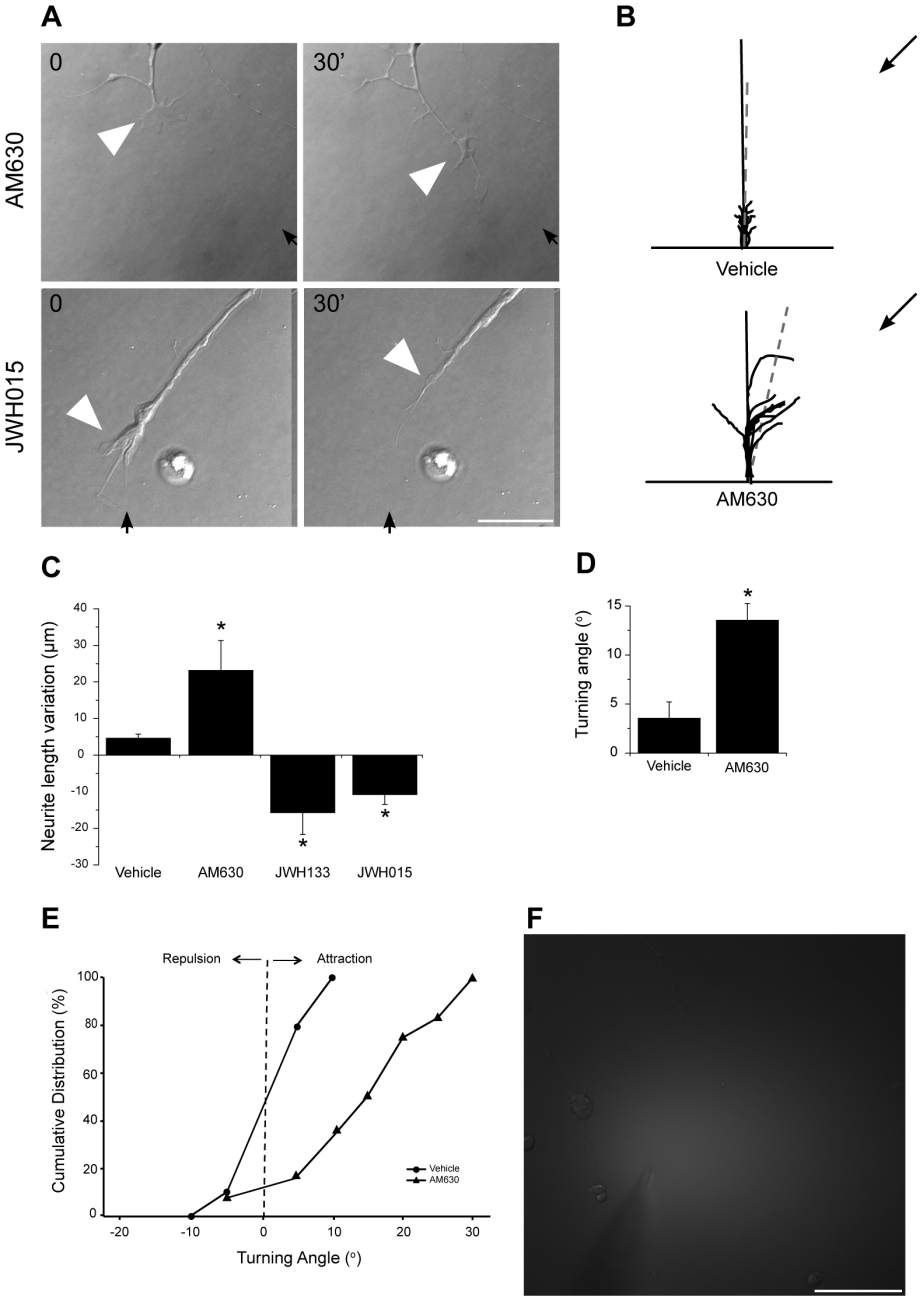
CB_2_R Agonists Influence Growth Cone Behavior. (A) Time-lapse microscopy DIV1 mouse retinal explant growth cone exposed to AM630 or JWH015 gradients. Arrows and arrowheads show micropipette angle and growth cone position, respectively. The micropipette tip diameter was 2–3 µm wide and positioned at 45° angle and 100 µm from the growth cone of interest. (B) Superimposed RGC axon trajectories over the 30 min observation period; black arrows indicate the direction of the gradient. Histograms illustrate neurite length (C) and turning angle (D) of growth cone following treatment (mean ± SEM; n = 7 to 13 per condition, * P<0.05 vs vehicle). (E) Turning angle cumulative frequency curves of RGC growth cones. The turning angle of each growth cone was plotted against the percentage of growth cones turning that angle or less. CB_2_R inverse agonist (AM630) increased axon growth and turning toward the pipette tip while CB_2_R agonists (JWH133 and JWH015) induced growth cone collapse, axon retraction. (F) Photomicrograph represents a microgradient created during drug application. Scale bars: 20 µm (A); 50 µm (F).

CB_1_R can mediate GABAergic interneurons growth cone repulsion *in vitro* [14]. Recently, we demonstrated that CB_1_R also modulates growth cone turning in glutamatergic neurons (RGCs) [15]. To evaluate whether CB_2_R is involved in retinal axon growth cone steering, turning assay experiments were performed on embryonic mice retinal cultures (Figure 7A). A microgradient application of JWH015 and JWH133 induced growth cone collapse and neurite retraction while AM630, elicited attractive turning (Figure 7 B–E). The vehicle (EtOH) did not induce any significant changes in growth cone direction. The concentration gradient was visualized with immunofluorescent dextran-FITC (Figure 7F), indicating that the drugs reached the growth cone. These results show that CB_2_R can modify axon growth and steering, and that its agonists can act as chemorepulsive signals on RGC growth cones.

### CB_2_R-Induced Growth Cone Morphological Changes Require PKA Activity

**Figure 8:**
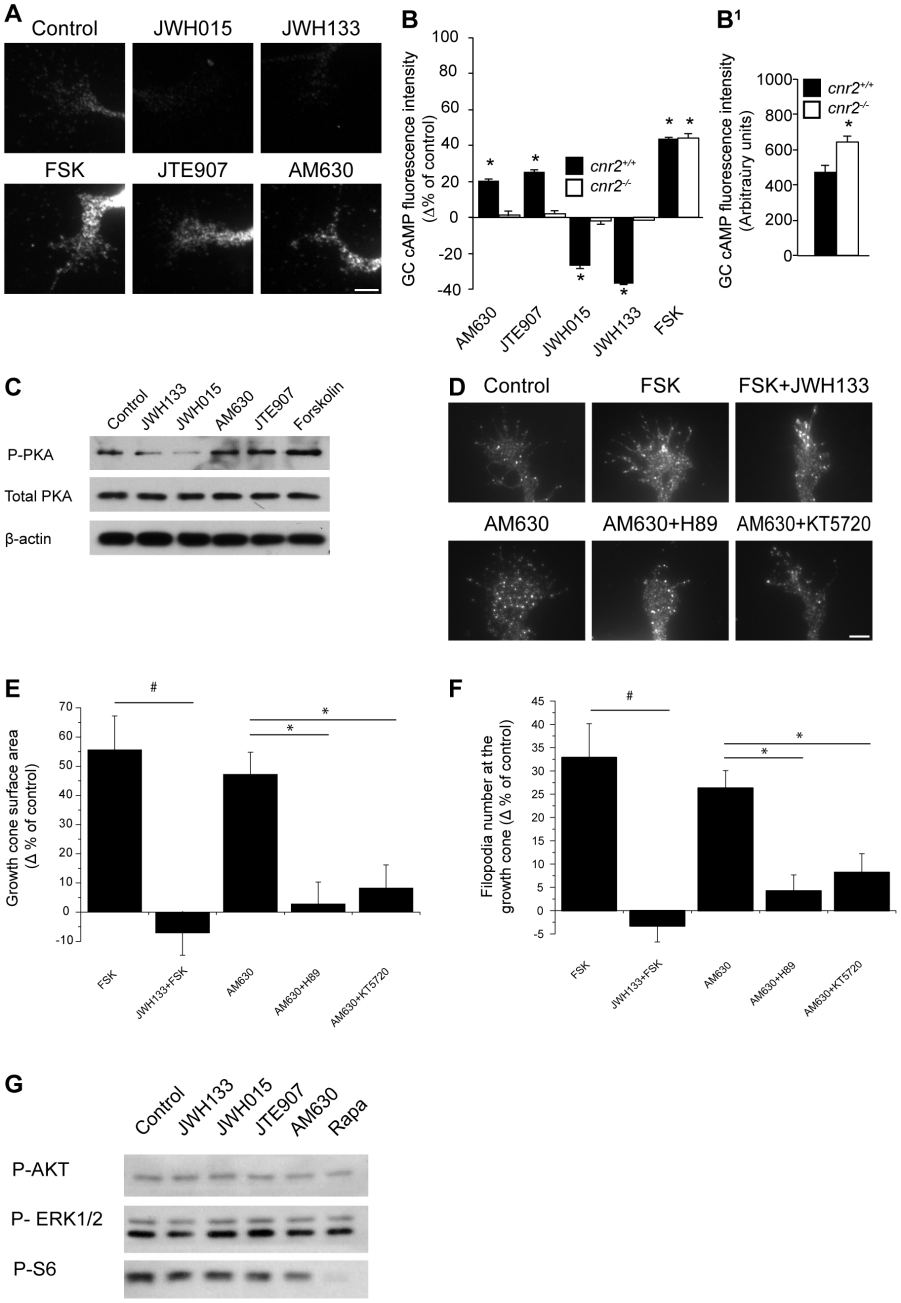
CB_2_R recruits the cAMP/PKA Pathway as a Downstream Effector. Growth cones were immunolabeled for intracellular cAMP following FSK, CB_2_R agonist (JWH015, JWH133), or CB_2_R inverse agonist (AM630, JTE907) application. (A) Representative photomicrographs. Scale bar, 5 µm. (B) Quantification of cAMP fluorescence intensity at the *cnr2^+/+^* growth cone indicates a significant decrease in cAMP levels following treatment with JWH133 and JWH015 while the opposite was observed following stimulation with AM630, JTE907 or FSK. No significative variations of cAMP were observed in growth cones obtained from cnr2^−/−^ after pharmacological treatments (mean ± SEM; n = 152 to 223 per condition, *P<0.05 vs control). (B^1^) Under control conditions, cAMP levels in growth cones of *cnr2^−/−^* embryos were significantly higher than those observed in wildtype embryos (mean ± SEM; n = 152 to 175 per condition, *P<0.05 vs control). (C) Western blot analysis indicates important changes in PKA phosphorylation levels following stimulation with CB_2_R agonists or inverse agonists. (D) For growth cone morphology analysis, neurons were exposed for 1 hour to FSK, FSK and JWH133, AM630, AM630 and H89 (a PKA inhibitor), or AM630 and KT5720 (another PKA inhibitor). Scale bar: 5 µm. Neurons were fixed and immunolabeled for GAP-43. Histograms represent quantification of growth cone surface area (E) and filopodia number (F). JWH133 abolished FSK induced increases in growth cone surface area and filopodia number and PKA inhibition abolished growth cone morphology modifications induced by AM630 (mean ± SEM; n = from 160 to 360 per condition, # P<0.05 vs FSK group *P<0.05 vs AM630 group). (G) Effect of the addition of CB_2_R agonists or inverse agonists on phosphorylation levels of AKT, ERK^½^ and S6 (P-AKT, P-ERK^1/2^ and P-S6).

Endocannabinoids and their CB_2_Rs have a diverse range of signal transduction mechanisms. Since it is well documented that stimulation of CB_2_Rs and subsequent activation of G_i/oα_ inhibits adenylate cyclase (AC) [34], we tested whether CB_2_R modulates the cAMP/PKA pathway during axon growth and guidance. We evaluated changes in growth cone intracellular cAMP levels following CB_2_R modulation using an antibody raised against cAMP (Figure 8A). In cnr2^+/+^, the CB_2_R agonists (JWH015 or JWH133) decreased cAMP levels at the growth cone as indicated by the lower fluorescence intensity compared with the control group (Figure 8A–B). Conversely, CB_2_R inverse agonists (AM630 or JTE907) as well as AC activator (forskolin) increased cAMP levels (Figure 8A–B). In cnr2^−/−^, CB2R agonists and inverse agonists did not produce any significant variation of cAMP levels. It is noteworthy that, under control conditions, deletion of *cnr2* signficantly increases growth cone cAMP level (Figure 8B ^1^). In another set of experiments, PKA phosphorylation was significantly lower following CB_2_R agonist stimulation while the opposite was observed following inverse agonist or FSK application as indicated by western blot analysis (Figure 8C). To further assess the implication of the cAMP/PKA pathway, primary neuron cultures were first treated with PKA-selective inhibitors followed by pharmacological manipulation of the CB_2_R. PKA inhibition (KT5720 or H89) blocked AM630-induced increases in growth cone surface area and filopodia number (Figure 8D–F). JWH133 abolished FSK-induced growth cone surface and filopodia increases (Figure 8D–F). These data demonstrate that CB_2_R activation modulates growth cone morphology via the cAMP/PKA pathway.

Upon activation, CB_2_Rs can also recruit other distinct second-messenger cascades including ERK1/2, PI3K/AKT and mTOR/S6K [35]–[37]. The implication of these signaling cascades was tested using western blot analysis. Interestingly, in primary neuron cultures, 10 min stimulation of the CB_2_R did not induce changes in AKT, ERK1/2, or S6 phosphorylation levels (Figure 8G). To validate these observations, the experiments were repeated for a 15 min stimulation period without any discernible activation of these pathways (data not shown). Altogether, these results demonstrate that the growth cone morphology reorganization induced by CB_2_R is dependent upon the cAMP/PKA pathway and not of ERK1/2, AKT or S6 signaling cascades.

### Deleted in Colorectal Cancer (DCC) Receptor is Required for CB_2_R Action on Growth Cone

Growth cone cytoskeleton reorganization in response to guidance cues is the main mechanism by which axons navigate toward their target cells [38]. Netrin is a chemotropic factor implicated in axonal guidance [39] and DCC, a transmembrane immunoglobulin superfamily receptor, is one of its receptors [40]. Growth cone morphology can be modified by DCC activation through substrate adhesion to netrin-1 and the recruitment of actin organization complex [41].

**Figure 9:**
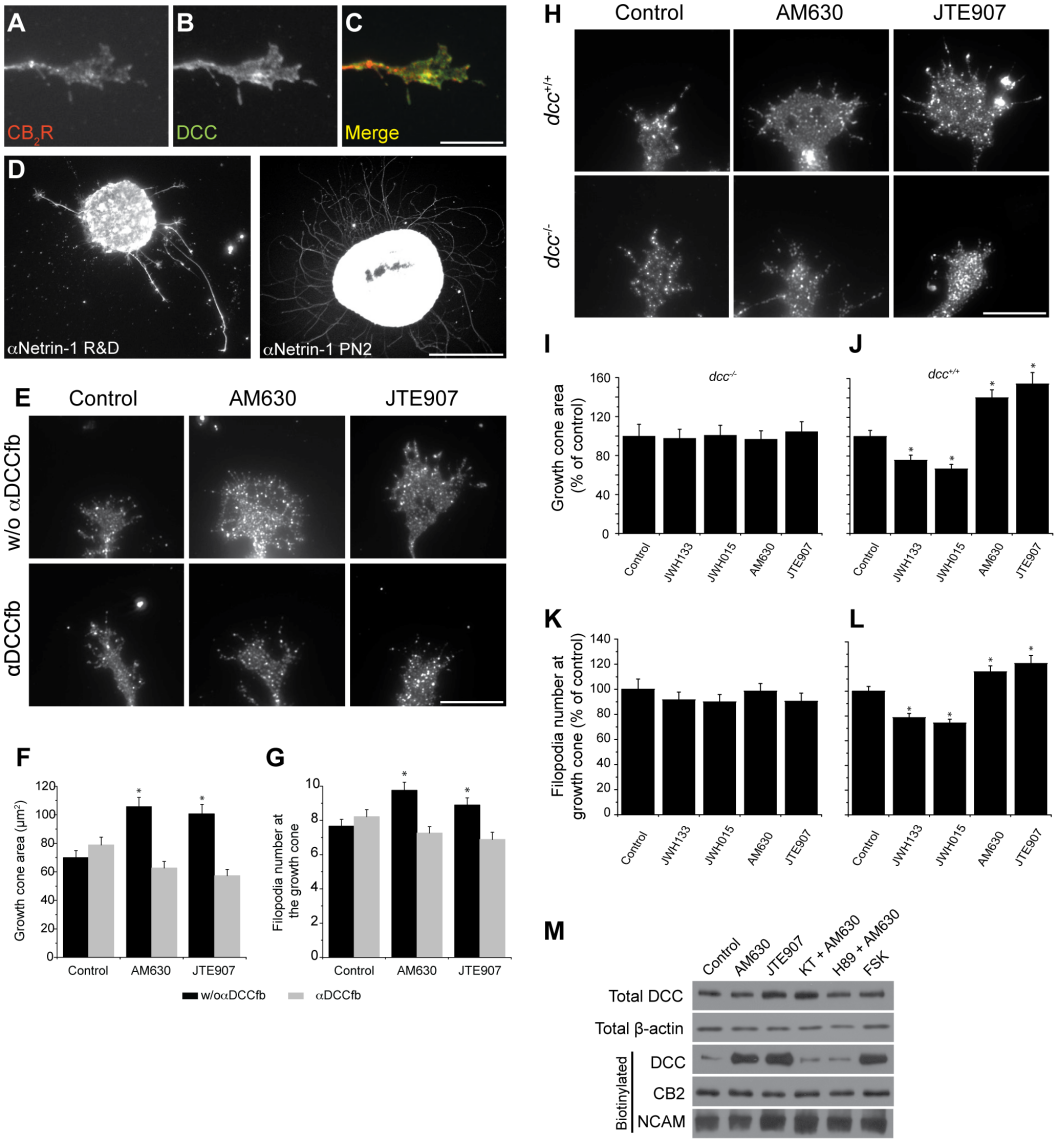
DCC Receptor is Required Downstream of PKA for CB_2_R Induced Reorganization of the growth cone. Retinal explants were grown for 1 DIV and growth cones were immunolabeled for CB_2_R (A) and DCC receptor (B). Merged image is presented in C. They were also immunostained for netrin-1, a ligand of DCC receptor (D). Dissociated neurons were cultured for 2 days *in vitro* and treated with pharmacological agents for 1 hour. (E) DCC function blocking (αDCC_fb_) antibody 3.5 µg/ml was added 15 minutes prior to AM630 or JTE907 stimulation. αDCCfb abolished AM630 and JTE907 induced increases in growth cone surface area (F) and filopodia number (G) (mean ± SEM; n = from 134 to 159 per conditions, *P<0.05 vs control condition). (H) Photomicrographs of growth cone for *dcc* ^+/+^ or *dcc* ^−/−^ mice. Histograms showing the size of growth cone area and the filopodia numbers in *dcc* ^+/+^ and *dcc* ^−/−^ animals (I–L). Pharmacological modulation of the CB_2_R did not induce any significant changes in growth cone surface area nor filopodia number in primary neuron cultures obtained from *dcc* ^−/−^ mice embryos (I and K) whereas JWH133 and JWH015 induced a decrease in growth cone surface and filopodia number while AM630 and JTE907 augmented these endpoints in *dcc* ^+/+^ neuron cultures (J and L) (mean ± SEM; 125 to 219 per conditions, *P<0.05 vs control condition). (M) DIV2 neurons were treated with AM630, JTE907, AM630 and KT5720, AM630 and H89, or FSK for 15 minutes. Following biotinylation and western blot, expression of surface protein was assessed for DCC receptor, CB_2_R and NCAM. Scale bar: 10 µm (A–C, E, H); 250 µm (D).

The cAMP/PKA pathway has been suggested to influence the growth cone’s sensitivity to netrin-1 [42]. In fact, activation of PKA increases netrin-dependent recruitment of DCC to the plasma membrane [43], [44]. Our results suggest that the cAMP/PKA pathway functions as a downstream effector for eCBs during growth cone guidance. Since CB_2_R, DCC, and netrin-1 are expressed in developing neurons (Figure 9A–D), it is reasonable to investigate the potential interactions between these molecules. DCC is widely expressed in RGCs [45] and cortical neurons [44] during development. Recently, we reported its presence in embryonic retinal explant axons, growth cones and filopodia [15]. Furthermore, DCC colocalizes with CB_2_R in these axons (Figure 9A–C). Hence, using pharmacological and genetic approaches, we investigated the potential interaction between these two receptors. First, we examined the effect of perturbing DCC function on CB_2_R evoked growth cone remodeling. Adding a DCC function-blocking antibody (αDCC_fb_) inhibits the CB_2_R inverse agonists AM630 and JTE907 induced-increase in growth cone surface area and filopodia number (Figure 9E–G). Secondly, CB_2_R agonists or inverse agonists did not induce any significant changes in growth cone area and filopodia number in neurons obtained from *dcc* ^−/−^ mouse embryos (Figure 9H–L). To determine whether CB_2_R activation modulates DCC trafficking to the plasma membrane, neurons were treated with AM630, JTE907, or FSK. Biotinylating cell surface proteins allowed the assessement of plasma membrane DCC. The relative amount of DCC present on the neuronal surface following the treatments was visualized by western blot analysis (Figure 9M). Interestingly, CB_2_R inverse agonists significantly increased the amount of DCC at the plasma membrane (Figure 9M). FSK also augmented the presence of DCC at the neuronal surface. To verify whether the CB_2_R induced DCC trafficking is upstream or downstream of PKA activation, neuronal cultures were treated with KT5720+AM630 or H89+AM630. As visualized by western blot, inhibition of PKA abolished AM630 induced increases in DCC at the plasma membrane (Figure 9M). Taken together, these results demonstrate that the CB_2_R induced reorganization of the growth cone implicates the presence of functional DCC receptors at the cell membrane.

### CB_2_R Modulates Retinal Projection Growth and Segregation *In Vivo*

Previous study on chick embryos have shown that inhibiting CB_1_R affects axonal growth [13]. Recently, we observed that CB_1_R modulates retinal projections *in vivo* [15]. In addition, retinal cAMP elevation was shown to increase retinal collateral length in the lateral terminal nucleus [28].

**Figure 10:**
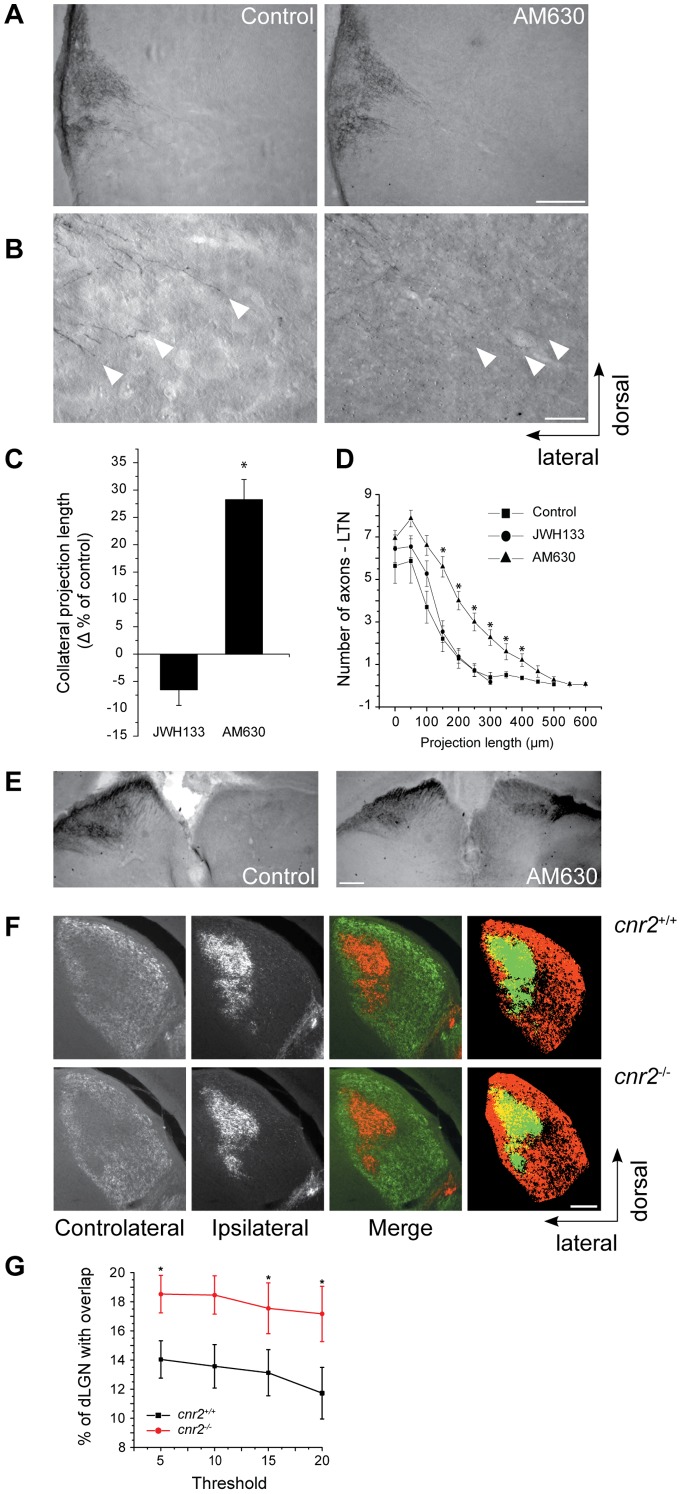
CB_2_R Modulates RGC Projection Development *In Vivo* and Eye-Specific Segregation in the Lateral Geniculate Nucleus. Hamsters at P1 were injected in the eye with CTb-FITC and 300 µM AM630, 300 µM JWH133 or vehicle control. Perfusion and brain fixation were done at P5. CTb revelation was performed with anti-CTb, enhanced with ABC Kit and revealed with DAB-Nickel kit. Photomicrographs of the lateral terminal nucleus (LTN) for the control and the AM630 groups (A) and terminal magnifications are shown (B). Quantification for collateral projection length is expressed as mean ± SEM percentage versus the control group (C). Quantification for axon branch density was also performed. AM630 increased axon growth (C) and collateral branch number (D) (n = 4 to 5 brains per condition, *P<0.05 versus control group). (E) Photomicrographs showing SC for the control and the treated groups. In the AM630 treated group, the presence of aberrant projections is illustrated by labeling in both hemispheres. (F) Fluorescence images of the dLGN for *cnr2* ^+/+^ and *cnr2* ^−/−^ mice showing contralateral projections from right eye injected with CTb-Alexa-546 and ipsilateral projections from left eye injected with CTb-Alexa-488. Merged images show all projections from both eyes to the dorsal lateral geniculate nucleus, overlaying projections are shown in yellow (F). (G) Graphic shows percentage of the dorsal lateral geniculate nucleus (dLGN) receiving overlapping inputs as mean ± SEM (n = 4 to 5 brains per condition, *P<0.05 versus control group). Quantification of the percentage of overlapping inputs in *cnr2* ^−/−^ and *cnr2* ^+/+^ adult mice indicating a significant increase in overlap between contralateral and ipsilateral RGC projections in the dLGN of *cnr2* ^−/−^ mice. Scale bars, 200 µm (A, F); 50 µm (B); 600 µm (E).

To assess the contribution of the CB_2_R pathway during the development of retinal projections *in vivo*, hamsters received intraocular injections of CB_2_R modulators. Compared to rats and mice, hamsters have shorter gestation period. Consequently, they are born with a relatively premature nervous system [24]. To take advantage of this opportunity, 24h after birth (P1), hamsters received a unilateral intraocular injection of AM630 or JWH133. Our data show that collateral projection length at the lateral terminal nucleus was significantly higher in the group treated with a CB_2_R inverse agonist when compared with the untreated group (Figure 10A–C). JWH133 did not affect significatively projection growth. Axon collateral density was also evaluated and branch density was significantly increased in the AM630-treated group (Figure 10D). In addition, interfering with the intrinsic ocular cannabinoid signaling with AM630 induced aberrant projections in the ipsilateral side of the SC as indicated by a robust labeling of retinal axons (Figure 10E).

During perinatal development, RGCs from both eyes send axons, which connect with multiple target cells in the dorsal lateral geniculate nucleus. These projections spread throughout the dLGN sharing common terminal space. During postnatal development, an eye-specific segregation occurs [46]. In the adult rodent, RGC axons occupy distinct eye-dependent non-overlapping regions of the dLGN. The implication of the CB_2_R during retinogeniculate development was further investigated in the dLGN in adult CB_2_R-deficient mice (*cnr2* ^−/−^) and their wild-type (*cnr2* ^+/+^) littermates. Adult *cnr2* ^+/+^ and *cnr2* ^−/−^ mice received a bilateral intra-ocular injection of the anterograde tracers CTb-Alexa488 in the left and of CTb-Alexa546 in the right eyes, respectively. Our data indicate a significant increase of the overlapping region between contralateral and ipsilateral RGC projections in the dLGN of *cnr2* ^−/−^ mice (Figure 10F–G). These observations confirm the essential role played by the CB_2_R during retinogeniculate development.

## Discussion

In the present study, we showed that CB_2_R is present throughout the visual pathway during development including *in vitro* primary RGC and retinal explants. CB_2_R activation modulates cAMP levels resulting in a PKA-dependent modification of the growth cone surface area and filopodia number. In addition, we observed that retinal axon outgrowth decreased following CB_2_R agonist treatment while stimulation with inverse agonist increased it. Most importantly, DCC, an axon guidance molecule receptor, is required for CB_2_R mediated morphological changes of the growth cone. *In vivo*, CB_2_R modulated RGC projection length, induced aberrant projections and the absence of this receptor altered eye-specific segregation. Taken together, these observations demonstrate that CB_2_R plays an essential role in the development of the retinothalamic pathway.

The expression of CB_2_R in the adult mammal brain has been previously detected by immunoreactivity [7], [8]. Although this receptor was also localized in developing neural progenitors and dorsal root ganglia [5], [6], its expression in retinal projections and along the visual tract remained unknown until now. Here, we show that RGC axons express CB_2_R and eCBs synthesis enzymes NAPE-PLD and DAGLα as well as degradation enzyme MGL. The receptor is present on axonal projections, growth cones and their filopodia *in vitro*. Moreover, *in vivo,* CB_2_R is localized at several important decision making points along the visual pathway. Indeed, CB_2_Rs are expressed in a spatio-temporal fashion in RGCs, optical chiasm, dLGN and SC. These results combined with those reported by Argaw et al (2011) clearly demontrate the presence of the eCB system in the developing neurovisual pathway and strongly suggest its influence on axonal navigation during CNS development.

In this study, CB_2_R effects on growth cone morphology were demonstrated in retinal axons and primary neuron cultures. Both bath application and microgradient stimulation studies showed its action on the growth cone. CB_2_R agonists produced chemorepulsive effect and collapse of the axonal growth cone. Additionally, CB_2_R had an important effect on retinal axon length for both short and long stimulation periods showing its capacity to influence axonal growth rate. These results are similar to those recently observed by our group for CB_1_R [15] and by other laboratories [13], [14], [47].

To characterize the mechanism by which CB_2_R modulates growth cone morphology and axonal growth, we examined an AC dependent signaling pathway [48]. Our study shows that CB_2_R acts by modulating intracellular cAMP concentrations, which directly influence PKA activity. These results are similar to those obtained with CB_1_R [15] but are in contradiction with a study showing that stimulation of CB_1_R with anandamide induced MAPK activation in GABAergic neurons [14]. However, under our experimental conditions, the MAPK signaling pathway was not modulated by CB_2_R. This could be explained in part by the fact that Berghuis et al. (2007) have studied the CB_1_R whereas we studied CB_2_R. Also, the difference in neuron types (GABAergic vs glutamatergic) could also account for the divergence in the downstream signaling pathways. We studied embryonic RGC growth cones that are glutamatergic in nature as compared to GABAergic interneurons favored by the Berghuis *et al*. (2007) study. Elevated cAMP levels in growth cone increase surface area and filopodia number in RGCs [28]. Adding CB_2_R inverse agonists to the neurons produced the same effect and increased growth cone cAMP levels. Furthermore, we showed that the CB_2_R modulates the cAMP/PKA pathway and that this signaling pathway is essential to the growth cone remodeling. This places PKA as an important downstream determinant for CB_2_R-induced growth cone reorganization.

PKA is not the only important molecule since DCC is also required for CB_2_R-induced growth cone morphology alterations. In the presence of an antibody that blocks DCC function, CB_2_R agonists or inverse agonists induced no changes in growth cone morphology. Furthermore, in *dcc* knockout mice, the absence of a functional DCC blocked the effects of CB_2_R on the growth cone. Earlier reports showed that PKA potentiated the mobilization of DCC to the cell surface [43], [44]. We have confirmed that elevation of PKA activity induces DCC receptor translocation to the plasma membrane through biotinylation analysis. Our results indicate that CB_2_R, which modulates cAMP levels, regulates growth cone expansion via a PKA-dependant mechanism. Variation of PKA activity will modulate the presence of DCC receptors at the growth cone surface and will induce growth cone morphological changes [43], [44]. A similar mechanism was observed with CB_1_R [15].

Recently, it was proposed that CB_1_R stimulation activates RhoA in GABAergic interneurons [14]. Spatially restricted activation of RhoA in the collapsing growth cone is associated with filopodia retraction and growth cone repulsion in response to chemical and electrical cues [49]–[51] through the activation of the serine-threonine kinase Rho kinase (ROCK) [51]. Moore et al. [52] demonstrated that RhoA inhibition recruits DCC to the plasma membrane. Therefore, CB_2_R agonists may, by increasing RhoA activity, prevent the presence of DCC at the membrane and consequently induce growth cone collapse. Conversely, CB_2_R inverse agonists or antagonists could decrease the activity of RhoA and promote axon growth via the translocation of DCC to the plasma membrane. Evidences from the literature suggest that there is an interaction between the RhoA and PKA pathways. In fact, PKA can directly inhibit RhoA [53]–[55], thus the PKA induced recruitment of DCC to the plasma membrane could result from several mechanisms and the inhibition of RhoA signaling might be one of them. We are currently investigating this possibility.

It is known that CB_1_R agonists induce neurite retraction in neuroblast cells [56] and chemorepulsive effect in GABAergic [14] and glutamatergic neurons [15]. Since CB_2_R can modulate growth cone morphology and its agonists have a repulsive effect on RGC axons, eCBs could act as an inhibitory signal in axon guidance. During brain development where axons travel relatively long distances to connect to specific neurons, CB_2_R could represent another mechanism by which eCBs modulate the guidance response to netrin-1 [42] or other guidance cues. In fact, integration of multiple cues by RGC axons may increase the specificity of their navigation and allow a better target recognition.

We showed that a single intraocular injection of AM630, a CB_2_R inverse agonist, increased the length of projections in the lateral terminal nucleus. Similar effects on growth rate were reported using cAMP analog [28]. We also observed the presence of aberrant ipsilateral RGC projections following a single intraocular injection of AM630. One may argue that the injection increased the branching or stabilized ipsilateral projections that would have normally retracted.

We noticed that adult *cnr2* ^−/−^ mice have increased overlapping regions of retinal projections from the two eyes in the dLGN compared to *cnr2* ^+/+^ mice. We interpreted this as a deficit in eye-specific segregation of retinal projections. In wildtype animals, this process could be influenced by eCB activity at the retina and/or directly at the axon terminal. It is possible that non functional CB_2_Rs influence retinal spontaneous activity, which is necessary for segregation and maintenance of specific inputs to the dLGN [57], thus modifying the segregation outcome. Deficiency in eye-specific segregation might also occur as a result of the absence of functional CB_2_R directly at the dLGN. In *cnr2* ^−/−^ mouse, eCBs would not be able to act as local modulators, inducing retraction of exuberant ectopic branches that have less activity. In fact, eCBs, via their action on CB_2_Rs may contribute to the normal inhibitory environment present in the CNS [58]. It is also reasonable to assume that *cnr2* knockout effect may not be as important as predicted because CB_1_R might in part surrogate CB_2_R activity, especially since main constituants of the eCBs, like 2-AG, have affinity for both receptors types [59], [60].

In conclusion, this study demontrates for the first time that CB_2_R is involved in axon guidance, and identifies the signaling pathway that mediates its effects. Therefore, we suggest a mechanism by which CB_2_R modulates retinothalamic development and nervous system wiring.
